# Infectivity of patent *Plasmodium falciparum* gametocyte carriers to mosquitoes: establishing capacity to investigate the infectious reservoir of malaria in a low-transmission setting in The Gambia

**DOI:** 10.1093/trstmh/trab087

**Published:** 2021-06-09

**Authors:** Abdullahi Ahmad, Harouna M Soumare, Muhammed M Camara, Lamin Jadama, Pa Modou Gaye, Haddy Bittaye, John Bradley, Jane Achan, Teun Bousema, Umberto D'Alessandro, Chris Drakeley, Marta Moreno

**Affiliations:** Medical Research Council Unit The Gambia at the London School of Hygiene and Tropical Medicine, Atlantic Boulevard, Fajara, PO Box 273, Banjul, The Gambia; Global Health Institute, Faculty of Medicine and Health Sciences, University of Antwerp, Doornstraat 331, 2610, Wilrijk, Belgium; Medical Research Council Unit The Gambia at the London School of Hygiene and Tropical Medicine, Atlantic Boulevard, Fajara, PO Box 273, Banjul, The Gambia; Medical Research Council Unit The Gambia at the London School of Hygiene and Tropical Medicine, Atlantic Boulevard, Fajara, PO Box 273, Banjul, The Gambia; Medical Research Council Unit The Gambia at the London School of Hygiene and Tropical Medicine, Atlantic Boulevard, Fajara, PO Box 273, Banjul, The Gambia; Medical Research Council Unit The Gambia at the London School of Hygiene and Tropical Medicine, Atlantic Boulevard, Fajara, PO Box 273, Banjul, The Gambia; Medical Research Council Unit The Gambia at the London School of Hygiene and Tropical Medicine, Atlantic Boulevard, Fajara, PO Box 273, Banjul, The Gambia; MRC International Statistics and Epidemiology Group, London School of Hygiene & Tropical Medicine, Keppel Street, WC1E 7HT, London, UK; Medical Research Council Unit The Gambia at the London School of Hygiene and Tropical Medicine, Atlantic Boulevard, Fajara, PO Box 273, Banjul, The Gambia; Radboud Institute for Health Sciences, Radboud University Medical Center, 6525 GA, Nijmegen, The Netherlands; Medical Research Council Unit The Gambia at the London School of Hygiene and Tropical Medicine, Atlantic Boulevard, Fajara, PO Box 273, Banjul, The Gambia; Department of Infection Biology, London School of Hygiene & Tropical Medicine, Keppel Street, WC1E 7HT, London, UK; Department of Infection Biology, London School of Hygiene & Tropical Medicine, Keppel Street, WC1E 7HT, London, UK

**Keywords:** *Anopheles coluzzii*, direct membrane feeding assays, gametocytes, *Plasmodium falciparum*, The Gambia

## Abstract

**Background:**

Understanding the human malaria infectious reservoir is important for elimination initiatives. Here, we implemented mosquito membrane feeding experiments to prepare for larger studies to quantify the transmission potential and relative contribution of the human infectious reservoir.

**Methods:**

Patients with clinical malaria attending four health facilities with at least 16 *Plasmodium falciparum* gametocytes per μL were recruited during the 2018 transmission season. Infectiousness to mosquitoes was assessed by direct membrane feeding assay (DMFA). We compared our results with a Bayesian predictive model to investigate the relationship between infectiousness and gametocyte density and explore the impact of fever on gametocyte infectivity.

**Results:**

A total of 3177 suspected malaria cases were screened; 43.3% (1376) had microscopically patent *P. falciparum* parasites and 3.6% (114) of them had gametocytes. Out of 68 DMFAs, 38 (55.9%) resulted in at least one infected mosquito, with a total of 15.4% (1178/7667) of mosquitoes infected with 1–475 oocysts per gut. The relationship between mosquito infection prevalence and gametocytaemia was similar to other African settings and negatively associated with fever (OR: 0.188, 95% CI 0.0603 to 0.585, p=0.0039).

**Conclusions:**

Among symptomatic malaria patients, fever is strongly associated with transmission failure. Future studies can use DMFA to better understand the human malaria reservoir in settings of low endemicity in The Gambia and inform malaria elimination initiatives.

## Introduction

Despite the extraordinary progress in reducing the global malaria burden achieved in the last 2 decades, progress towards elimination has stalled in recent years.^1^ Malaria transmission has not been interrupted despite high coverage of standard control intervention such as insecticide-treated bed nets and novel tools may be needed to further reduce transmission.^2^

The successful transmission of the infection from the human host to the mosquito vector depends on the presence of infectious gametocytes in the human peripheral blood.^3^ These gametocytes, when ingested by mosquitoes during the blood meal, can develop into ookinetes, oocysts and, finally, into sporozoite stages in the salivary glands, rendering these mosquitoes infectious to humans in subsequent blood meals. Transmission to mosquitoes is associated with gametocyte density^4^ but also with less clearly parametrised factors such as gametocyte maturity,^5^ sex ratio^6,7^ and human characteristics including immune factors^8^ and potentially inflammatory markers associated with clinical malaria episodes.^9^

The assessment of malaria transmission potential depends on mosquito-feeding experiments that typically allow mosquitoes to feed on blood from infected individuals.^10,11^ The most frequently used assays are the direct skin feeding assay and direct membrane feeding assay (DMFA), with the former resulting in slightly higher mosquito infection rates.^12^ The difference between DMFA and direct skin feeding assay is probably not due to gametocyte sequestration in skin tissue,^13,14^ as long hypothesised,^15^ but rather to technical challenges that prevent premature gametocyte activation after phlebotomy^16^ and anticoagulants used in blood collection.^17^ The use of DMFA, instead of direct skin feeding assay, allows investigating infectivity in all age groups, using larger numbers of mosquitoes to be examined per experiment and the direct quantification of gametocyte densities in the blood meal used.^18^ DMFA experiments should be standardised, as slight variations in experimental procedures may have important implications for the experiments’ outcome and complicate comparisons between different studies.^11^

The Gambia has been extremely successful in reducing its malaria burden over the last 20 y thanks to scaling up malaria control interventions.^19^ However, there is still significant residual malaria in the eastern part of the country,^20^ maintained by a substantial human reservoir of infection, with many low-density infections.^21^ Quantifying the transmission potential of the human reservoir of infection is important to support elimination initiatives; more specifically, the contribution of low-density infections to onward transmission currently remains unknown.^22,23^ This requires sensitive DMFA experiments.

In the current study, we implemented mosquito membrane-feeding assays in the Upper River Region (URR), the region in The Gambia contributing to most malaria cases, and upon which we rely for future epidemiological studies assessing the human malaria reservoir. The success of the experiments was assessed by comparing infectiousness and mosquito infectivity rates when feeding on patent *Plasmodium falciparum* gametocyte carriers. Also, we contrasted our results with those previously obtained in a predictive model on gametocyte infectiousness in relation to gametocyte density. Finally, we evaluated the association of fever and other factors to the infectiousness of local gametocyte carriers.

## Methods

### Screening and enrolment

The study was conducted in the URR in eastern Gambia, an area with low to moderate malaria transmission (13.1%, *P. falciparum* prevalence by PCR^21^) and with the highest incidence of clinical malaria in the country, that is, 0.8–1.0 *P. falciparum* episodes per person-year at risk.^20^ From September 2018 to January 2019, patients with suspected malaria attending four health facilities (Basse Hospital and Sabi, Sotuma Sere, Gambissara health centres) were screened for eligibility. Patients aged ≥2 y had a blood sample collected for thick blood films. Microscopy-positive cases with a *P. falciparum* gametocyte count of at least 1 gametocyte per 500 white blood cells (WBC) (i.e. 16 gametocytes/μL), regardless of asexual parasite count, were enrolled in the study after providing written informed consent and, where applicable, written assent. Severe malaria patients and those with a history of sulfadoxine-pyrimethamine and/or amodiaquine use in the previous 7 d were excluded. Parasite density per microlitre was estimated by assuming 8000 WBC per microlitre.

### Insectary

The *Anopheles coluzzii* colony was established in our insectary in May 2018 with eggs acquired from Institute Pasteur de Dakar, Senegal. The insectary building is divided into four working areas for larvae rearing (one room, 4×3 m^2^, temperature 39°C and relative humidity [RH] 70±10%), adult maintenance (one room for colony and for infectious mosquito storage, 4×3 m^2^, temperature 27±2°C and RH 70±10%) and two additional spaces for feeding experiments (3×2 m^2^, temperature 27±2°C and RH 70±10%) and mosquito dissections (4×3 m^2^, ambient temperature). Adult mosquitoes were maintained with 10% glucose solution and Hemotek system (Hemotek Ltd, Blackburn, UK) was used for blood feeding and eggs production. The yield of adult mosquitoes per week is 10 000 with the capacity to conduct up to five independent membrane feeding experiments per day. The access of the insectary is restricted to users (by PIN keypad) and electric flycatcher traps are located in any potential exit routes from the insectary.

### Membrane feeding

Enrolled participants were accompanied within 1 h of enrolment by a health worker to the insectary at the Medical Research Council Unit, The Gambia, field station in Basse for DMFA experiments. A study nurse closely monitored symptoms in readiness to provide immediate treatment if needed; otherwise participants were treated with artemether-lumefantrine immediately after phlebotomy, in line with the national guidelines for treating uncomplicated malaria in The Gambia. An additional thick smear was prepared immediately before mosquito feeding to identify any differences in gametocyte density since screening.

At the insectary, venous blood samples were collected in lithium-heparin 4-mL tubes (BD Vacutainer Franklin Lakes, NJ, US). Immediately after phlebotomy, three aliquots of 400–500 μL of whole blood were transferred to glass feeders and approximately 50 female *A. coluzzii* mosquitoes (aged 3–6 d) per feeder (150 in total) were allowed to feed through a parafilm membrane for 20 min. In preparation for these experiments, mosquitoes were starved overnight (minimum 8 h).^24^ All the mosquito-feeding experiments were conducted from 10:00 h to 16:00 h. Partially fed and non-fed mosquitoes were removed and only fully blood-fed mosquitoes were kept in the insectary at 27±2°C, 70±10% RH and 12L:12D photoperiod (12 h of light and 12 h of darkness per day), with access to glucose solution until dissection, for 7–8 d postblood meal. Mosquito midguts were dissected and microscopically examined to assess the presence of oocysts (mercurochrome stained 0.5%); mosquito infection results were recorded for separate feeders/cups to allow analysis of variability between feeders.

### Sample size calculation

This study was a preparatory study to ensure that mosquito-feeding assay performance justified a larger study on the human infectious reservoir of infection. We calculated the sample size on the assumption that at least half of the patients with microscopy-detectable gametocytes would infect ≥1 mosquito if each experiment examined approximately 50 mosquitoes.^12^ This is in line with more recent assessments of gametocyte density by gametocyte-specific *Pfs25* mRNA by qRT-PCR and the relationship with mosquito infectiousness.^4^ Recruiting 50 patients with a gametocyte density of at least 16/μL would provide at least 25 infectious individuals (95% CI 23 to 51% and thus including the expected 50%), assuming a total of at least 10% infected mosquitoes.

### Statistical analysis

The main study outcomes were the proportions of infected mosquitoes (mosquitoes with ≥1 oocyst) and infectious individuals (individuals who infected at least one mosquito). Baseline measures of parasitaemia and gametocytaemia and the distribution of parasites by age were compared using a non-parametric Kruskal– Wallis test. A mixed effects logistic regression model using the lme4 package via the glmer function in R v. 3.0.2 (Vienna, Austria) was used to investigate the association of age (used as a categorical variable to be consistent with our previous assessments of infectivity), gender, fever (≥37.5°C), date of the mosquito-feeding experiment, asexual parasitaemia (log-transformed) and gametocytaemia (log-transformed) with infectiousness. This was done at the level of the mosquito, with infection status as the dependent variable and a random effect for the study participant. To further assess the relationship between transmission and gametocyte density, the curve obtained from a Bayesian predictive model, using Bayesian Markov Chain Monte Carlo methods, was fitted and a graph was drawn to check if the relationship was similar to previously observed results.^4^

## Results

From September 2018 to January 2019, a total of 3177 suspected malaria cases were screened for eligibility and 43.3% (1376) had a confirmed *P. falciparum* infection. About half of them (50.8%, 688/1354) were females and the median age of patients with *P. falciparum* infection was 12 y (IQR: 7–20) (Table 1). Asexual parasite density was similar between children aged <5 y (4000 parasites/μL; IQR: 800–14 160) and >5 y (upper limit 15 y) (4160 parasites/μL, IQR: 800–12 000) but significantly lower in adults (640 parasites/μL, IQR: 0–3520) (p=0.0104) (Table 1).

**Table 1.** Summary of participant characteristics

	Asexual parasite carriers^1^ (n=1354)	Gametocyte carriers^2^ (n=114)
Age (in y), median (IQR)	12 (7–20)	10 (6–15)
Age group (in y), n (%)		
2 to 5	131 (9.7)	18 (15.8)
>5 to 15	704 (51.9)	67 (58.8)
>15	519 (38.3)	29 (25.4)
Gender, n (%)		
Male	666 (49.2)	66 (57.9)
Asexual parasites per μL, median (IQR)	7520 (1440–28 640)	2640 (520–7480)
Asexual parasites per μL per age group (in y), median (IQR)		
2 to 5	7360 (1360–33 840)	4000 (800–14 160)^a^
>5 to 15	13 280 (2080–37 320)	4160 (800–12 000)^b^
>15	4320 (1280–15 680)	640 (0–3520)^c^
Gametocyte density per μL, median (IQR)	-	64 (32–192)
Gametocyte density per μL per age group (in y), median (IQR)		
2 to 5	-	80 (40–200)
>5 to 15	-	64 (32–240)
>15	-	32 (16–268)

^1^All the asexual parasite carriers were included regardless of the presence of gametocytes. Individuals without asexual parasites detected by microscopy (n=21) were not included in this category.

^2^All gametocyte carriers identified in the health facilities were included.

^a,b,c^ Differences between asexual parasite carriers and gametocyte carriers were statistically significant (p<0.001).

Gametocyte prevalence by microscopy among all parasite carriers was 8.3% (114/1376); 18.4% (21/114) participants carried only gametocytes (i.e. no asexual stages) (Table 1). Gametocyte prevalence decreased with age: 13.3% (18/135) of children aged <5 y, 9.4% (67/713) in the 5–15 y age group and 5.5% in adults (29/527). Children aged <5 y tended to have higher gametocyte density (80 gametocytes/μL, IQR: 40–200) than children aged 5–15 y (64 gametocytes/μL, IQR: 32–240) and adults (32 gametocytes/μL, IQR: 16–268) (p=0.654) (Table 1).

Overall, gametocyte density increased significantly as the transmission season progressed (median gametocytes/μL: 26, 74, 296 and 474 in September [n=4], October [n=23], November [n=39] and December [n=43], respectively; p=0.034).

### Membrane feeding experiments

Out of the 114 patients detected with gametocytes at the health centres, 19 declined to participate in the study, 4 had complicated malaria (one of the exclusion criteria), 9 participants were not included due to exceeding the insectary capacity at the moment of recruitment and 9 could not be conducted due to mosquito husbandry issues. Finally, for 5 participants there was no parent or guardian present to sign the informed consent (e.g. minors accompanied to the health facility by siblings or other family member with no custody), therefore, they were not recruited for the study.

Of the 68 DMFA experiments performed, more than half of the participants infected at least one mosquito (55.9%, 38/68) (Table 2). Overall, mosquito feeding rate (94.2%, 9610/10 200) and survival until the day of dissection were high (79.8%, 7667/9610). Of all mosquitoes analysed, 15.4% were infected with *P. falciparum* (1178/7667) with a geometric mean of 2 oocysts per mosquito (range 1–475 oocysts/midgut) and a mean of 114 mosquitoes per participant were dissected (range 72–139). From the feedings with at least one infected mosquito per experiment (n=38), oocyst data were grouped by feeder (one, two or three) and no significant differences were detected in the number of oocysts per mosquito between the three groups of feeders (Kruskal–Wallis rank-sum test, p=0.55).

**Table 2.** Summary of membrane feeding experiments and infectivity by age group

	Age group, in y, n				
	2–5 (n=7)	>5–15 (n=40)	>15 (n=21)	All ages (n=68)	p
Asexual parasite density per μL, median (IQR)	1120 (400–9520)	4480 (1200– 145 600)	1440 (0–4160)	3040 (480–8960)	0.096^b^
Gametocyte density per μL, median (IQR)	128 (40–208)	120 (32–256)	32 (16–224)	80 (28–244)	0.537^b^
Fever (≥37.5°C), %	57.1 (4/7)	37.5 (15/40)	42.9 (9/21)	41.1 (28/68)	0.106^c^
Infectious individuals, %	57.1 (4/7)	62.5 (25/40)	42.9 (9/21)	55.9 (38/68)	0.147^c^
Infected mosquitoes, %	8.2 (67/815)	17.9 (804/4495)	13.0 (307/2357)	15.4 (1178/7667)	0.170^c^
Infected mosquitoes, %^a^	14.3 (67/470)	29.0 (804/2774)	30.2 (307/1016)	27.7 (1178/4260)	0.039*^,c^
Mean infection intensity (range)	3.5 (1–18)	9.1 (1–475)	2.3 (1–25)	4.9 (1–475)	0.317^b^
Dissected mosquitoes per feed, median (IQR)	120 (111.5–130.5)	112 (109–118.5)	114 (108–118)	114 (109–120)	0.860^c^

^a^Median, only data from infectious individuals included; ^b^Kruskal–Wallis test to assess between-group difference; ^c^χ^2^ test to assess between group difference.

*p<0.05.

The relationship between gametocytaemia and mosquito infectiousness was further investigated. Opposite to gametocyte density distributions in different age groups, mosquito infection rate was significantly lower in children aged <5 y (14.3%) than in older children (29.0%) and adults (30.2%) (p=0.039) (Table 2). As expected, gametocyte densities were higher when slides were read specifically for enumeration in the insectary compared with initial screening at facilities to simply identify the presence of gametocytes (Wilcoxon signed rank-sum test, p<0.001).

**Figure 1.**
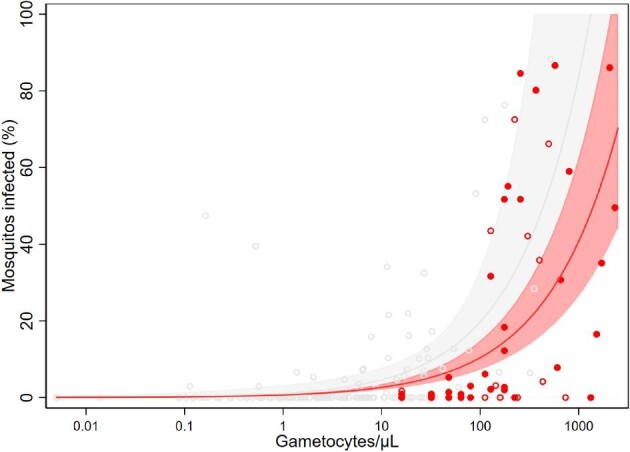
Relationship between total gametocyte density and the percentage of mosquitoes that develop oocysts. Red dots are observations from this study: open red dots correspond with febrile participants and solid red dots with non-febrile participants; the solid red line indicates the best-fit model, with the light red shaded area indicating the 95% CI around this line. Grey open dots correspond to observations from a previous study^4^; the solid grey line indicates the best-fit model, with the light grey shaded area indicating the 95% CI around this line.

Infectiousness varied by month, between 33% in September and 52% in December, and peaked in November (71%). The proportion of infected mosquitoes increased rapidly beyond 100 gametocytes per microlitre (Figure 1). At the time of recruitment, 41.2% (28/68) participants had fever (axillary T≥37.5°C) (median 37.2°C, IQR: 36.8–37.9°C). Mosquito infectiousness was negatively associated with fever in the regression model (OR: 0.188, 95% CI 0.0603 to 0.585, p=0.0039) and no significant association was detected with the other covariates in the regression model (age, gender, gametocytaemia, parasitaemia or the date of infection).

## Discussion

Membrane-feeding assays are an essential tool to study factors influencing malaria transmission and to identify and quantify the human infectious reservoir. The use of these assays in the field requires a standardisation of procedures to minimise variability and mimic natural transmission conditions.^12,24^ In this study, we established DMFAs in an area with low-moderate transmission in The Gambia and we qualified the experiments by assessing the mosquito infectivity of *P*. falciparum gametocytes from patent symptomatic infections. The probability of a mosquito becoming infected after feeding on symptomatic *P. falciparum* gametocyte carriers’ blood was >55%, a figure similar to those (58.2% and 60.7%) observed earlier in Farafenni, The Gambia.^25,26^ In another study in Senegal with similar transmission intensity, >44% of the patent gametocyte carriers (aged ≥5 y) infected at least one mosquito.^27^ A wide range of mosquito infectiousness has been recorded across different transmission intensities. For example, in Burkina Faso, the proportion of children aged <15 y with patent gametocytaemia infecting at least one mosquito was 68.2%^28^; other studies have reported rates ranging from 74 to 84%.^29^ Conversely, infectivity of *P. falciparum* in other low endemic non-African settings, such as Cambodia, can be significantly lower, with up to 6.2% of patent infections successfully transmitted to mosquitoes.^30^

Our study is in line with previous findings where prevalence of gametocytaemia was greatest in patients with lower asexual parasite density.^31^ Lower asexual parasite density has been identified as one predictor of gametocytaemia and may reflect chronic infections more likely to have circulating gametocytes. About 40% of gametocyte carriers in our study had fever at recruitment. Considering the delay in gametocyte production upon incident infection, it is possible that either gametocytes arose from an earlier infection (and fever at the time of sampling was caused by a more recent malaria infection) or that there was a non-malaria cause of fever in the presence of gametocytes. Gametocyte infectiousness was lower among gametocyte carriers with confirmed fever (T≥37.5°C) at the moment of mosquito feeding, as shown elsewhere.^32^ This apparent decrease in mosquito infection rates has been associated with cytokine-mediated inactivation of gametocytes.^9^

In our study, the higher infectiousness in children aged <5 y and school-age children (5–15 y) compared with older individuals (>15 y) was consistent with younger children being generally more infectious to mosquitoes.^33^

Consistent with other studies, we observed a positive correlation between gametocyte density and mosquito infection.^4,12^ However, some individuals with high gametocyte densities did not infect any mosquitoes.^12,34^ Gametocyte carriage or density alone cannot predict mosquito infectivity, and highlight the need to directly quantify the transmissibility of infections. Such assessments of gametocyte infectiousness have been predominantly performed in areas of intense malaria transmission.^35^ The human infectious reservoir remains particularly poorly understood in areas approaching elimination, where low-density infections are prevalent.^33^

This study included only symptomatic individuals, thus it does not represent the whole malaria transmission spectrum in the study area. In addition, individuals with subpatent gametocytaemia were probably excluded as no molecular analysis for its detection was carried out. Nevertheless, our sampling strategy was designed to demonstrate the implementation of DMFAs for measuring the infectivity of natural gametocyte carriers in comparison with previous studies.^12^ DMFA implementation is limited by the required short time between blood collection and mosquito feeding to ensure gametocyte infectivity.^36^ This issue can be logistically solved by bleeding infected individuals close to the insectary, although this may discourage study participation, or by developing procedures to transport blood samples (e.g. stable environmental variables such as temperature) from the place of bleeding to the insectary.^37^

As transmission declines in The Gambia, the proportion of infections with low parasite density may increase. Having successfully implemented DMFAs will enable further research to understand the contribution of low-parasite density infections to residual transmission in an area near to elimination such as The Gambia.^23,38^

### Conclusions

Assessing the human malaria infectious reservoir is crucial for elimination initiatives. This study yielded results in line with other studies and prior associations of gametocyte density and mosquito infection by DMFAs. Thus, the assay can be used in future studies to quantify the transmission potential and evaluate the impact of transmission-blocking interventions.

## Data Availability

The data underlying this article will be shared on reasonable request to the corresponding author.

## References

[1] Bhatt S , Weiss DJ, Cameron E et al. The effect of malaria control on *Plasmodium falciparum* in Africa between 2000 and 2015. Nature. 2015;526(7572):207–11. 26375008 10.1038/nature15535 PMC4820050

[2] WHO . World Malaria Report 2019. Geneva, Switzerland: World Health Organization, 2019.

[3] Rabinovich RN , Drakeley C, Djimde AA et al. malERA: an updated research agenda for malaria elimination and eradication. PLoS Med. 2017;14(11):e1002456. 29190300 10.1371/journal.pmed.1002456 PMC5708604

[4] Bradley J , Stone W, Da DF et al. Predicting the likelihood and intensity of mosquito infection from sex specific *Plasmodium falciparum* gametocyte density. Elife. 2018;7:34463. 10.7554/eLife.34463 PMC6013255 29848446

[5] Bousema T , Drakeley C. Epidemiology and infectivity of *Plasmodium falciparum* and *Plasmodium vivax* gametocytes in relation to malaria control and elimination. Clin Microbiol Rev. 2011;24(2):377–410. 21482730 10.1128/CMR.00051-10 PMC3122489

[6] Reece SE , Drew DR, Gardner A. Sex ratio adjustment and kin discrimination in malaria parasites. Nature. 2008;453(7195):609–14. 18509435 10.1038/nature06954 PMC3807728

[7] Tadesse FG , Meerstein-Kessel L, Goncalves BP et al. Gametocyte sex ratio: the key to understanding *Plasmodium falciparum* transmission? Trends Parasitol. 2019;35(3):226–38. 30594415 10.1016/j.pt.2018.12.001 PMC6396025

[8] Bousema T , Sutherland CJ, Churcher TS et al. Human immune responses that reduce the transmission of *Plasmodium falciparum* in African populations. Int J Parasitol. 2011;41(3-4):293–300. 20974145 10.1016/j.ijpara.2010.09.008 PMC3052432

[9] Naotunne TS , Karunaweera ND, Mendis KN et al. Cytokine-mediated inactivation of malarial gametocytes is dependent on the presence of white blood cells and involves reactive nitrogen intermediates. Immunology. 1993;78(4):555–62. 8495973 PMC1421895

[10] Sauerwein RW , Bousema T. Transmission blocking malaria vaccines: assays and candidates in clinical development. Vaccine. 2015;33(52):7476–82. 26409813 10.1016/j.vaccine.2015.08.073

[11] Miura K , Stone WJ, Koolen KM et al. An inter-laboratory comparison of standard membrane-feeding assays for evaluation of malaria transmission-blocking vaccines. Malar J. 2016;15:463. 27612458 10.1186/s12936-016-1515-z PMC5016893

[12] Bousema T , Dinglasan RR, Morlais I et al. Mosquito feeding assays to determine the infectiousness of naturally infected Plasmodium falciparum gametocyte carriers. PLoS One. 2012;7(8):e42821. 22936993 10.1371/journal.pone.0042821 PMC3425579

[13] Meibalan E , Barry A, Gibbins MP et al. *P. falciparum* gametocyte density and infectivity in peripheral blood and skin tissue of naturally infected parasite carriers in Burkina Faso. J Infect Dis. 2019;jiz680. 10.1093/infdis/jiz680 PMC8161640 31875909

[14] Talman AM , Ouologuem DTD, Love K et al. Uptake of *Plasmodium falciparum* gametocytes during mosquito bloodmeal by direct and membrane feeding. Front Microbiol. 2020;11:246. 32194521 10.3389/fmicb.2020.00246 PMC7062676

[15] Van Den Berghe LCM , Peel E. Supériorité des preparations de scarification du derme sur les préparations de sang périphérique pour le diagnostic de malaria. An Inst Med Trop. 1952(9):553–62. 13058140

[16] Graves PM. Studies on the use of a membrane feeding technique for infecting *Anopheles gambiae* with *Plasmodium falciparum*. Trans R Soc Trop Med Hyg. 1980;74(6):738–42. 7010696 10.1016/0035-9203(80)90189-3

[17] Solarte Y , Manzano Mdel R, Rocha L et al. Effects of anticoagulants on *Plasmodium vivax* oocyst development in *Anopheles albimanus* mosquitoes. Am J Trop Med Hyg. 2007;77(2):242–5. 17690393

[18] Bousema T , Churcher TS, Morlais I et al. Can field-based mosquito feeding assays be used for evaluating transmission-blocking interventions? Trends Parasitol. 2013;29(2):53–9. 23273727 10.1016/j.pt.2012.11.004

[19] Ceesay SJ , Casals-Pascual C, Erskine J et al. Changes in malaria indices between 1999 and 2007 in The Gambia: a retrospective analysis. Lancet. 2008;372(9649):1545–54. 18984187 10.1016/S0140-6736(08)61654-2 PMC2607025

[20] Mwesigwa J , Achan J, Di Tanna GL et al. Residual malaria transmission dynamics varies across The Gambia despite high coverage of control interventions. PLoS One. 2017;12(11):e0187059. 29095834 10.1371/journal.pone.0187059 PMC5667860

[21] Mwesigwa J , Slater H, Bradley J et al. Field performance of the malaria highly sensitive rapid diagnostic test in a setting of varying malaria transmission. Malar J. 2019;18(1):288. 31455349 10.1186/s12936-019-2929-1 PMC6712604

[22] Okell LC , Bousema T, Griffin JT et al. Factors determining the occurrence of submicroscopic malaria infections and their relevance for control. Nat Commun. 2012;3:1237. 23212366 10.1038/ncomms2241 PMC3535331

[23] Slater HC , Ross A, Felger I et al. The temporal dynamics and infectiousness of subpatent Plasmodium falciparum infections in relation to parasite density. Nat Commun. 2019;10(1):1433. 30926893 10.1038/s41467-019-09441-1 PMC6440965

[24] Ouédraogo AL , Guelbéogo WM, Cohuet A et al. A protocol for membrane feeding assays to determine the infectiousness of *P. falciparum* naturally infected individuals to *Anopheles gambiae*. Malar World J. 2013;4(16):1–4. 10.5281/zenodo.10926272 PMC11138739 38828116

[25] Drakeley CJ , Akim NI, Sauerwein RW et al. Estimates of the infectious reservoir of *Plasmodium falciparum* malaria in The Gambia and in Tanzania. Trans R Soc Trop Med Hyg. 2000;94(5):472–6. 11132369 10.1016/s0035-9203(00)90056-7

[26] Drakeley CJ , Secka I, Correa S et al. Host haematological factors influencing the transmission of *Plasmodium falciparum* gametocytes to *Anopheles gambiae* s.s. mosquitoes. Trop Med Int Health. 1999;4(2):131–8. 10206267 10.1046/j.1365-3156.1999.00361.x

[27] Gaye A , Bousema T, Libasse G et al. Infectiousness of the human population to *Anopheles arabiensis* by direct skin feeding in an area hypoendemic for malaria in Senegal. Am J Trop Med Hyg. 2015;92(3):648–52. 25624409 10.4269/ajtmh.14-0402 PMC4350567

[28] Ouedraogo AL , Bousema T, Schneider P et al. Substantial contribution of submicroscopical *Plasmodium falciparum* gametocyte carriage to the infectious reservoir in an area of seasonal transmission. PLoS One. 2009;4(12):e8410. 20027314 10.1371/journal.pone.0008410 PMC2793432

[29] Hien AS , Sangare I, Coulibaly S et al. Parasitological indices of malaria transmission in children under fifteen years in two ecoepidemiological zones in southwestern Burkina Faso. J Trop Med. 2017;2017:1507829. 28286526 10.1155/2017/1507829 PMC5327772

[30] Vantaux A , Samreth R, Piv E et al. Contribution to malaria transmission of symptomatic and asymptomatic parasite carriers in Cambodia. J Infect Dis. 2018;217(10):1561–8. 29394367 10.1093/infdis/jiy060

[31] WWARN Gametocyte Study Group . Gametocyte carriage in uncomplicated Plasmodium falciparum malaria following treatment with artemisinin combination therapy: a systematic review and meta-analysis of individual patient data. BMC Med. 2016;14:79. 27221542 10.1186/s12916-016-0621-7 PMC4879753

[32] Barry A , Bradley J, Stone W et al. Higher gametocyte production and mosquito infectivity in chronic compared to incident Plasmodium falciparum infections. Nat Commun. 2021;12(1):2443. 33903595 10.1038/s41467-021-22573-7 PMC8076179

[33] Goncalves BP , Kapulu MC, Sawa P et al. Examining the human infectious reservoir for Plasmodium falciparum malaria in areas of differing transmission intensity. Nat Commun. 2017;8(1):1133. 29074880 10.1038/s41467-017-01270-4 PMC5658399

[34] Churcher TS , Bousema T, Walker M et al. Predicting mosquito infection from *Plasmodium falciparum* gametocyte density and estimating the reservoir of infection. Elife. 2013;2:e00626. 23705071 10.7554/eLife.00626 PMC3660740

[35] Stone W , Goncalves BP, Bousema T et al. Assessing the infectious reservoir of falciparum malaria: past and future. Trends Parasitol. 2015;31(7):287–96. 25985898 10.1016/j.pt.2015.04.004

[36] Da D , Lefevre T, Yerbanga RS et al. Mosquito direct membrane feeding assay: overcome the field constraints and adapt the method for the evaluation of malaria transmission-blocking interventions. Am J Trop Med Hyg. 2017;97(5):310.

[37] Soumare HM , Guelbeogo WM, van de Vegte-Bolmer M et al. Maintaining *Plasmodium falciparum* gametocyte infectivity during blood collection and transport for mosquito feeding assays in the field. Malar J. 2021;20(1):191. 33879163 10.1186/s12936-021-03725-y PMC8056727

[38] Collins KA , Ouedraogo A, Guelbeogo WM et al. Investigating the impact of enhanced community case management and monthly screening and treatment on the transmissibility of malaria infections in Burkina Faso: study protocol for a cluster-randomised trial. BMJ Open. 2019;9(9):e030598. 10.1136/bmjopen-2019-030598 PMC6747640 31519680

